# Bivalent Surface Attachment via Cysteine Thiol Results in Efficient and Stereoselective Abiotic Peptide Synthesis

**DOI:** 10.1021/jacsau.5c00153

**Published:** 2025-03-31

**Authors:** Daniel P. Molland, Isabella B. Rhyu, Jon Wade, Jason R. Schnell

**Affiliations:** † Department of Biochemistry, University of Oxford, South Parks Road, OX1 3QU Oxford, U.K.; ‡ Department of Earth Sciences, University of Oxford, South Parks Road, OX1 3AN Oxford, U.K.

**Keywords:** origin of life, abiogenesis, peptide synthesis, cysteine, silicate minerals

## Abstract

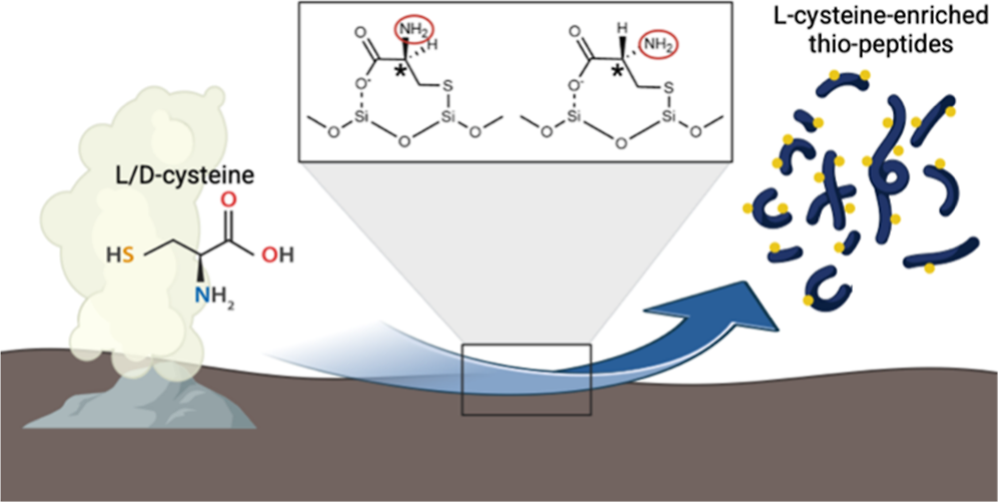

Surface-catalyzed peptide bond formation may have been an important source of peptides for abiogenesis, but model peptide synthesis reactions using the consensus set of 10 abiotic amino acids give only modest rates of peptide bond formation. Additionally, the peptides are typically limited in length to a small number of amino acids and stereoselective amino acid incorporation is weak or absent. An abiotic route for the high-yield synthesis of cysteine from serine was recently reported by Foden et al. (*Science***2020**, 370, 865–869), indicating that, in some environments, prebiotic cysteine may also have been available. Here, we show that the presence of cysteine dramatically increases the yields of surface-catalyzed peptide synthesis reactions in a hydrothermal vent solvent model containing achiral silicate minerals and that the reaction exhibits a strong stereoselective bias toward l-cysteine. Solid state NMR confirmed that cysteine associates bivalently with silicates at alkaline pH via both the carboxylate and the sulfur groups. Polarization-resolved IRRAS indicates that the bivalent adsorption stereospecifically orients the reactive amino group, providing a mechanism for stereoselective incorporation of l-cysteine. Stereoselective rates of peptide bond formation in surface-catalyzed peptide bond formation are expected to occur for any amino acid able to form sufficiently strong side chain–silicate interactions at alkaline pH. The high nucleophilicity of the thiol group produces unusually high reaction rates and stereoselectivity in such reactions, in addition to conferring transition metal ion binding to the peptide products. The potential benefits of reactive sulfur species for abiogenesis suggest that they may be useful biosignatures in the search for habitable extraterrestrial environments.

## Introduction

Proteins are essential components of life, but their prebiotic origins remain unresolved.^1^ Carbon-rich meteorites contain up to 2300 ppm of amino acids and are proposed to have been a major source of the early Earth’s inventory of organic molecules.^2,3^ Present in carbonaceous chondrites, the amino acids glycine, alanine, aspartate, glutamate, valine, isoleucine, leucine, serine, threonine, and proline constitute the proteogenic amino acid set.^4,5^ This proteogenic set of amino acids is restricted to neutral or negatively charged side chains and is limited in functionality due to their low side chain nucleophilicity.^6^

Several mechanisms have been proposed for synthesis of peptides from this set, including salt induced peptide formation^7^ and activating agent catalyzed peptide synthesis.^8,9^ The best explored of these synthetic routes is surface-catalyzed peptide synthesis, first proposed by Bernal et al.,^10^ and since documented under a wide range of reaction conditions^11^ and minerals.^12^ Mineral surfaces catalyze peptide synthesis by binding amino acids, typically through electrostatic interactions, and increasing local amino acid concentrations.^11,12^ The extent to which a particular surface can enhance peptide synthesis is driven predominantly by residence times on the mineral surface, which depend on the strength of the interaction and electrostatics that affect diffusion near the surface.^12^

The condensation reaction to form peptide bonds is facilitated at alkaline pH by deprotonation to form the more nucleophilic NH_2_ group.^13^ Moderate reaction temperatures are optimal,^14,15^ since diketopiperazines are formed at higher temperatures (≥120 °C).^16^ These observations point to alkaline hydrothermal vent systems, like the white smokers of the Atlantis Massif, as suitable environments for prebiotic peptide synthesis.^17^ The Enceladian ocean is proposed to contain similarly alkaline and moderately hot ocean vents.^18−20^

While peptide synthesis from amino acids can be catalyzed by high salt, metals, and surfaces, peptide bond yields are typically low and peptide lengths typically limited to ∼2–6 amino acids.^17,21−24^ In surface-catalyzed peptide synthesis, this limitation is a consequence of the relatively weak association between peptides and the mineral surface and the formation of peptide-chain terminating end products such as piperazines.^21^ In addition, stereoselectivity is typically low or absent. A <10% preference for l-amino acids was observed for metal-mediated catalysis in high salt conditions,^25^ although the mechanism was not identified.^26^ Demonstrations of higher stereoselectivity required chiral templating minerals or chiral biotic precursors.^27,28^ Thus, none of the currently described synthetic processes provide a general mechanism for high-yield and stereoselective synthesis of longer peptides.^17^

Here, we investigated the potential role of highly nucleophilic thiol in abiotic peptide synthesis in a surface-based reaction mechanism. We found that the addition of cysteine in the presence of a silicate surface increased peptide synthesis 7.8-fold, producing peptides as long as 12 amino acids in length, and that peptide yields were 50% stereoselective for l-cysteine over d-cysteine. Our results are consistent with an unusually strong stereospecific silicate association mechanism for cysteine. We show that both cysteine enantiomers strongly attach to the SiO_2_ surface through both the carboxylate and the highly nucleophilic sulfur group simultaneously, which results in the amino group being stereospecifically oriented in the surface-bound state. We propose that this orientation-dependent effect from the bivalent attachment is primarily responsible for the observed rate enhancement.

This work points to an efficient mechanism for stereoselective peptide bond formation through bivalent surface attachment under alkaline reducing conditions. The increased abiotic yield of thiol-containing peptides, which have metal binding capability,^29,30^ indicate that thiol-bearing amino acids like cysteine or homocysteine could be essential ingredients for abiogenesis.

## Results

### Increased Peptide Synthesis Yields and Stereoselectivity with Cysteine

A reaction scheme was established based on the oxide mineral and amino acid reaction systems of Takahagi et al.^16^ All reaction mixtures contained a racemic meteoric amino acid mixture (MAAM) supplemented with an additional l- or d-amino acid at excess concentration to test its effect on peptide yields. Consistent with surface-catalysis, reactions in borosilicate vessels led to 1.8-fold increases in peptide yields over reactions in polypropylene vessels due to the additional silicate surface area (Figure S1a).

**Figure 1:**
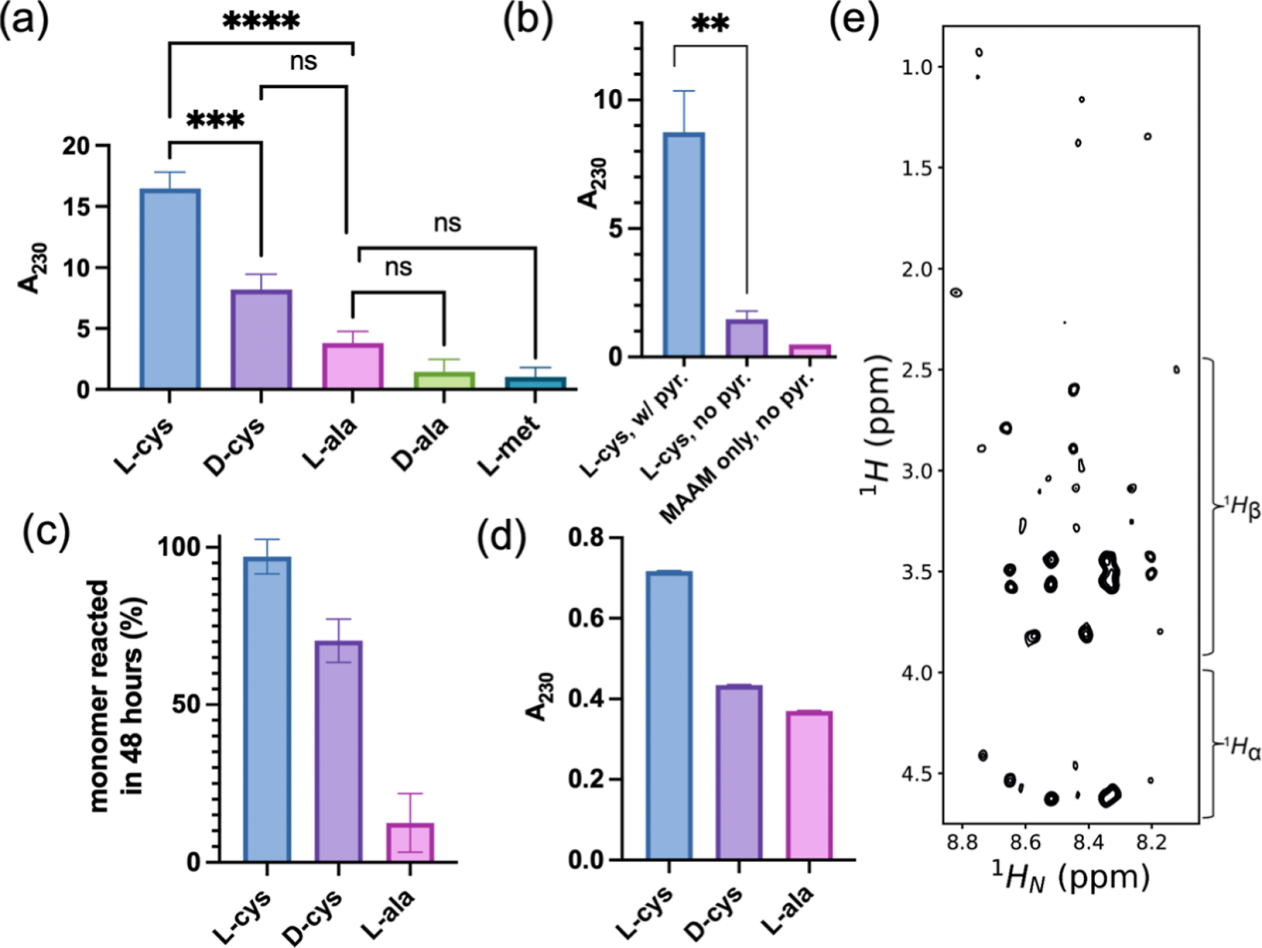
Cysteine stereospecifically increases peptide yield in the presence of an achiral oxide mineral. (a) Detection of peptide bond formation by absorbance at 230 nm (A_230_) after 48 h of heating in borosilicate reaction vessels with MAAM plus an excess of l-cysteine (*n* = 6), d-cysteine (*n* = 5), l-alanine (*n* = 6), d-alanine (*n* = 3), or l-methionine (*n* = 2). Biological repeats were normalized to the A_230_ with l-alanine. Normalization relative to l-cysteine was used to determine the standard error of the mean (SEM; error bars) of l-alanine biological repeats. The mean control absorbance of l-alanine, d-alanine and l-methionine was 2.10. Significance was determined by one-way ANOVA with Tukey posthoc analysis of absorbance vs l-alanine (l-cysteine, *p* = <0.0001; d-cysteine, *p* = 0.085; d-alanine, *p* = 0.712; l-methionine, *p* = 0.702) (Table S1). (b) A_230_ amino acid mixtures reacted in polypropylene reaction vessels with 5 mM l-cysteine, 5 mM MAAM, and 0.5 g pyroxene (l-cys, w/pyr.; *n* = 3), 5 mM l-cysteine, 5 mM MAAM, and no pyroxene (l-cys, no pyr.; *n* = 3), and 10 mM MAAM with no l-cysteine or pyroxene (MAAM only, no pyr.; *n* = 1). For the comparison of reactions containing l-cysteine and with or without pyroxene, *p* = 0.0015 (two-tailed unpaired *t* test) (Table S2). Error bars indicate SEM; (c) loss of monomeric amino acid reactant by qNMR with a benzoic acid internal standard after 48 h of reaction time. Error bars display standard deviation of the benzoic acid signal height at 7.4034 ppm. (d) A_230_ measurement of stereoselective peptide bond formation following reactions at lower (55 μM) amino acid starting concentrations. Reactions were heated at 90 °C for 42 days in borosilicate reaction vessels. Error bars indicate the standard deviation for technical repeats (*n* = 3). (e) 2D ^1^H–^1^H TOCSY spectrum of a postreaction mixture enriched in l-cysteine. The spectral region corresponds to intraresidue correlations between amide protons (^1^HN) and either ^1^Hα (∼4.0–4.7 ppm) or side chain protons (∼1.0–4.0 ppm).

Initial assessment of peptide yield was conducted by UV-spectrophotometry (Table S1).^31^ In borosilicate reaction vessels, absorption of 230 nm light (A_230_) increased significantly after 48 h in the presence of l-cysteine compared to reactions with either l-alanine (*p* = <0.0001) or d-cysteine (*p* = 0.0006). In contrast, synthesis with excess l- or d-alanine (*p* = 0.7172) and l-alanine or l-methionine (*p* = 0.7021) did not result in significantly increased peptide yields, indicating that these enantiomers absorbed to silicates similarly at alkaline pH. When compared to the mean absorbances for l-alanine, d-alanine, and l-methionine, the presence of l-cysteine increased peptide bond yield 7.8-fold (±0.6), and d-cysteine increased absorbance 3.9-fold (±0.6) (Figure 1a).

The presence of silicate surfaces significantly amplifies the catalytic effect of cysteine on the peptide bond formation. In the absence of a silicate surface, peptide bond yields were increased approximately three-fold in the presence of cysteine, consistent with a previous report,^32^ and the addition of a silicate surface (pyroxene) increased this value another 6-fold (*p* = 0.0015; two-tailed unpaired *t* test) (Figure 1b and Table S2). The apparent rates of reaction after 48 h for systems containing pyroxene and either l-cysteine, d-cysteine or l-alanine in 5 mM excess were 0.76, 0.38, and 0.12 mM of peptide bonds per hour, respectively.

Given the large number of organic species that could have been generated in our model system with absorbance at A_230_, reaction products were further investigated by ^1^H–^1^H and ^1^H–^13^C solution nuclear magnetic resonance (NMR). Quantitative NMR (qNMR) measurements after 48-h reactions with either l-cysteine or l-alanine indicated that 7.7-fold more monomeric l-cysteine was removed than l-alanine (Figure 1c). The stereoselective bias toward l-cysteine persisted at the lowest concentrations of amino acids tested (55 μM; Figure 1d). ^1^H–^1^H total correlation spectroscopy (TOCSY) of the reacted mixtures revealed signals consistent with diverse ^1^H_N_–^1^H_α_ and ^1^H_N_–^1^H_side chain_ correlations that were not observed in the unreacted control samples (Figure 1e) and confirmed that the increases in A_230_ seen in l- and d-cysteine correlated with the formation of peptide bonds.

### Peptide Synthesized with Cysteine are Compositionally Diverse

**Figure 2:**
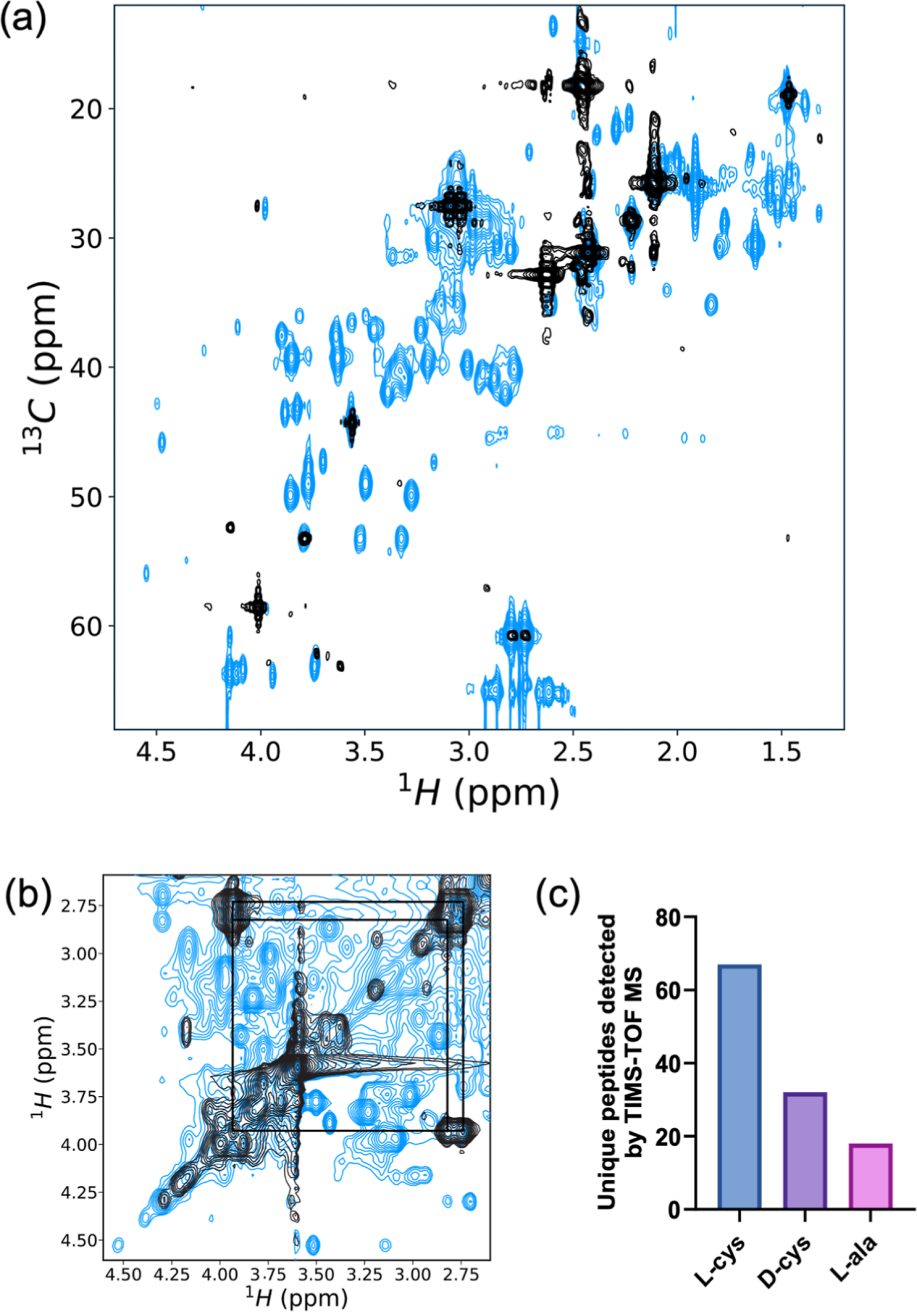
Peptide synthesis with cysteine yields a diverse set of cysteine-containing peptides. (a) Overlay of 2D ^1^H–^13^C HSQC spectra of reactions containing ^13^C_β_-labeled l-cysteine before (black) and after (blue) reaction. New crosspeaks in the postreaction spectrum are attributed to incorporation of ^13^C_β_-labeled l-cysteine into peptides adjacent to different amino acid types. Additional crosspeaks arise from natural abundance ^13^C (1.1%). (b) Overlay of 2D ^1^H–^1^H TOCSY spectra of reaction mixtures containing l-cysteine before (black) and after (orange) reaction. The intense cysteine monomer ^1^H_α_–^1^H_β_ crosspeaks observed before reaction are indicated by lines. The large number of new postreaction crosspeaks are attributed to unique peptide species. (c) Histogram of the total number of unique peptide products in l-cysteine reactions identified by TIMS–TOF MS after purification by SPE and HPLC prior to MS loading.

NMR spectra of products formed in the presence of ^13^C_β_l-cysteine after heating at 90 °C for 48 h indicated a large number of unique signals in the chemical shift range expected for nonoxidized cysteine ^1^H_β_–^13^C_β_ and ^1^H_α_–^13^C_α_ correlations, as well as amide ^1^H to ^1^H_α_ and ^1^H_β_, which were consistent with the formation of a diverse set of cysteine-bearing peptides (Figure 2a,b). Inspection of the prereaction solutions confirmed no major contaminants were present in the solution prior to synthesis. In contrast to the reactions with cysteine, few unique signals were observed when synthesis was conducted in the presence of ^13^C-labeled l-methionine (Figure S2).

Trapped ion mobility spectrometry time-of-flight (TIMS-TOF) mass spectrometry (MS) of 42-day reactions (90 °C) detected the largest number of unique peptides from reactions containing l-cysteine (Figure 2c). The number of unique peptides detected was likely underestimated due to the requirement for a C18 purification before MS that enriches the sample in hydrophobic peptides (Figure S3). In reactions with l-cysteine, significantly larger numbers of unique peptides longer than four amino acids were detected compared to reactions with l-alanine (Mann–Whitney Test, *p* = 0.0188) (Table S3). In reactions with l-cysteine, peptides containing up to 12 amino acids were detected, whereas the largest peptide detected in reactions with l-alanine contained 8 amino acids. An increase in the number of detectable peptides was also observed with d-cysteine compared to l-alanine but not to statistical significance (*p* = 0.1563) (Figure S4 and Table S4). The increased detections of peptides in reactions with l-cysteine compared with d-cysteine or l-alanine were consistent with the yields determined from A_230_ and qNMR, and indicated a stereoselective bias of higher peptide bond formation in the presence of l-cysteine.

### Increased Peptide Yields with Cysteine Are Due to the Thiol

The increased peptide yields with l- and d-cysteine compared to l-alanine correlate with the high nucleophilicity of the side chain thiol,^6^ which results in strong attachment to silicate surfaces. Reactions in the presence of l-homocysteine showed a level of peptide bond formation comparable to that of l-cysteine (Figure S1b), whereas l-methionine showed no statistically significant increase in peptide bond formation compared to l-alanine (Figure 1b). Reaction yields with excess l-aspartate were intermediate between l-alanine and l-cysteine (Figure S1b), suggesting that silicate binding via the side chain carboxylate group can also contribute to increased yields although this interaction is weaker due to the lower nucleophilicity of the carboxylate compared with the thiol.^6,33^

A high correlation between the peptide bond yield and the corresponding metal–sulfur bond dissociation energies was observed in the presence of different metal oxides, consistent with the strength of attachment being the dominant factor affecting peptide bond yield (Figure S5). The magnitude of the trend did not extend to nonmetallic silicates and the very high silicate-sulfur dissociation energy, possibly indicating that adsorption was no longer rate-limiting.^34^

### Bivalent and Stereospecific Adsorption of Cysteine at Alkaline pH

**Figure 3:**
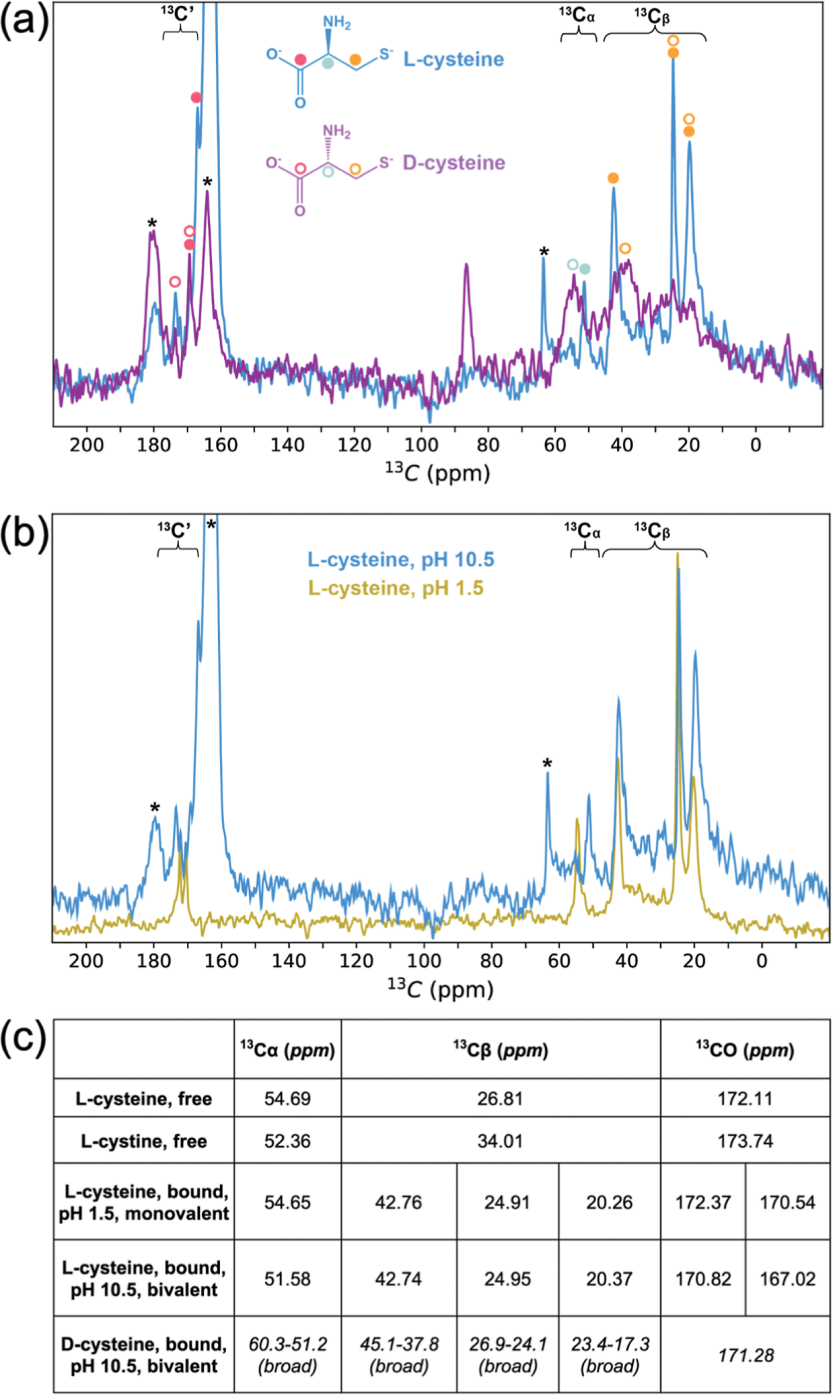
d-cysteine and l-cysteine association with silicates is bivalent at alkaline pH. (a) Overlay of solid-state NMR ^13^C cross-polarization spectra of l-cysteine (blue) and d-cysteine (purple) adsorbed to silicate at pH 10.5. The ^13^C resonances are indicated by colored circles for l-cysteine (closed) and d-cysteine (open). Chemical shift positions of the enantiomers are similar for carboxylate ^13^C′ signals and differ for the ^13^Cα and ^13^Cβ signals. Wash buffer signals at 163.2 ppm (bicarbonate), 169.3 ppm (carbonate) 179.7 ppm (carbonate salt), and 63.7 ppm (bicarbonate sideband) are indicated (*). (b) Solid-state NMR ^13^C cross-polarization spectra of l-cysteine adsorbed to silicates at pH 10.5 (blue) and pH 1.5 (yellow). The ^13^Cβ signals are independent of pH, whereas the ^13^Cα and carboxylate ^13^C′ signals are perturbed at low pH consistent with de/protonation of the carboxylate. (c) ^13^C chemical shifts determined from solid-state spectra. Asterisks indicate the observed chemical shifts consistent with covalent thiolate attachment.

The binding of cysteine to silicate surfaces through both the sulfur and carboxylate groups was confirmed by measuring the NMR chemical shifts of the silicate-bound cysteine. Solid state ^13^C cross-polarization spectra of cysteine could be recorded despite extensive washing of the silicate with cysteine-free solution (Figure 3a). The ^13^C_α_ and ^13^C_β_ chemical shifts of both l-cysteine and d-cysteine were strongly perturbed from that of unbound cysteine or cystine (Figure S6), indicating that both enantiomers bound strongly to the silicate. The apparent line widths of the d-cysteine ^13^C_α_ and ^13^C_β_ peaks were much larger than those of l-cysteine, indicating changes in dynamics or the presence of multiple states for surface-adsorbed d-cysteine. d-cysteine also exhibited an additional peak at ∼85 ppm, which could not be assigned. The identical solution state chemical shifts of the cysteine enantiomers were confirmed (Figure S7), indicating that the unique solid state chemical shifts were due to stereospecific interactions at the silicate surface.

The downfield shifted ^13^C_β_ signals of l-cysteine and d-cysteine were consistent with the chemical shift reported previously for cysteine bound to gold,^35^ reflecting the formation of covalent silicothio ether bonds (−Si–S–C(H_2_)−). The presence of this covalent bond was confirmed by polarization resolved infrared reflection absorption spectroscopy (PR-IRRAS) on samples of l-cysteine adsorbed to silicate films, which showed an absorption band for p-polarized light at 1407 cm^–1^ (Figure S8),^36^ similar to that measured for a Si–S–CH_2_ bond in silicon bound ethanethiol (1406 cm^–1^).^37^ Two additional resonances for ^13^C_β_ could be detected upfield of the covalent silicothioether signal for both l- and d-cysteine, indicating the presence of noncovalent, electrostatically bound complexes with the silicate surface. In contrast, single resonances were detected for ^13^C_α_ of silicate-bound cysteines.

The presence of attachment via the carboxylates was tested by recording spectra of l-cysteine at a low enough pH to cause carboxylate protonation and weakening of any silicate interactions. The l-cysteine ^13^C_β_ chemical shifts were similar at pH 1.5 and pH 10.5 and strongly perturbed compared with free cysteine, indicating that silicate attachment via the sulfur occurs over a wide pH range. In contrast, the l-cysteine ^13^C_α_ chemical shift at pH 1.5 (54.65 ppm) was similar to that of silicate-free l-cysteine (54.69 ppm), and the intense, upfield shifted carboxylate ^13^C resonance observed at pH 10.5 (170.82 and 167.02 ppm) was replaced by two downfield shifted resonances (172.37 and 170.54 ppm), suggesting that carboxylate protonation had weakened or altered a carboxylate–silicate interaction. Thus, cysteine attaches bivalently through both the side chain sulfur and main chain carboxylate at high pH but is attached predominantly monovalently via a side chain sulfur at low pH.

### Stereospecific Orientation of the Amino Group in Bivalently Silicate Adsorbed Amino Acids

Although the chemical shifts of both d- and l-cysteine were strongly shifted relative to those of the free amino acids, the ^13^C_α_ and ^13^C_β_ (covalently bound form) chemical shifts of d- and l-cysteine were distinct, suggesting that they adopt unique conformations at the silicate surface. The different physical properties that arise from bivalent surface attachment of enantiomers were investigated further by PR-IRRAS, which provides bond angle information for surface adsorbed molecules.

**Figure 4:**
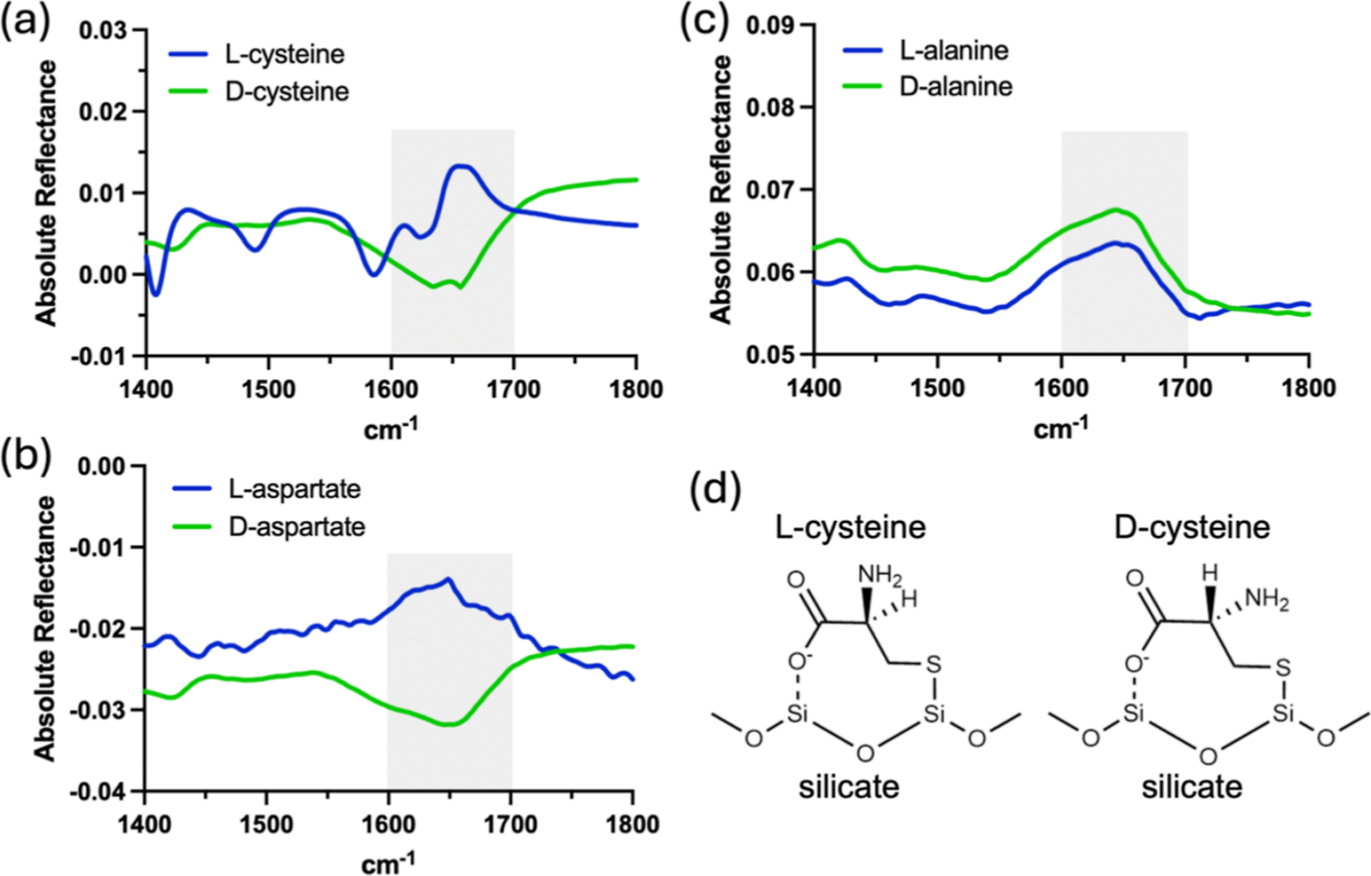
Bivalent surface attachment stereospecifically orients the amino acid amino group. PR-IRRAS of the enantiomers of (a) cysteine, (b) aspartate, and (c) alanine adsorbed to silicate. Stereospecific inversion is observed in the reflectance corresponding to the asymmetric bend of the amino group (∼1600–1700 cm^–1^) in samples containing cysteine and aspartate but not alanine. Full spectra are shown in Figure S9. (d) Schematic of bivalently attached l- and d-cysteine indicating the proposed stereospecific orientation of the amino group.

PR-IRRAS of amino acids adsorbed to Ti/Au/SiO_2_ microfilm slides showed reflectance in p-polarized light but not s-polarized light, consistent with the signals arising from surface-bound amino acids. The reflectance bands for the amino group (N–H) asymmetric bend were inverted between the cysteine enantiomers, and for the aspartate enantiomers (Figure 4a,b),^38^ indicating stereospecific differences in the orientations of the amino groups for cysteine and alanine when bound to silicate. In contrast, no inversion of this amino group reflectance band was detected for the alanine enantiomers (Figure 4c). The observation of such a stereospecific reorientation of the amino group for cysteine and aspartate is consistent with their ability to attach bivalently through both the main chain carboxylate and side chain (thiol or carboxylate, respectively) (Figure 4d). Stereospecific reorientation of the reactive amino group provides a possible mechanism for stereoselective differences in the rate of peptide bond formation.

## Discussion

A growing body of work suggests the importance of sulfur to early life (reviewed in ref (39)), but the effects of having a reactive sulfur-bearing amino acid like cysteine in abiotic surface-catalyzed peptide synthesis systems had not been investigated previously.^40,41^ All 10 amino acids in the conventional set of abiotic amino acids are able to interact with silicates via the main chain carboxylate group, but this interaction is weak, and amino acids with nonreactive side chains are easily washed from silicates.^42^ The only reactive side chain chemical groups in the abiotic set of 10 are the carboxylates of aspartate and glutamate and the hydroxyls of serine and threonine, which only modestly increase silicate surface adsorption. The recent discovery of a potential pathway toward abiotic synthesis of cysteine opens up the possibility that its dramatically more nucleophilic thiol side chain may have facilitated surface-catalyzed peptide bond formation.^6,32^ We show here that the addition of cysteine to mixtures of the otherwise conventional set of 10 abiotically available amino acids significantly increases peptide bond yields when heated in the presence of achiral silicate minerals at alkaline pH. The formation of a covalent silicothioether bond was easily detectable by NMR after extensive washing of the cysteine–silicate complex, and the peptide bond yields correlated with the strength of the sulfur bond to metal oxides, demonstrating that the increase in yields with cysteine are attributable to the increased strength of surface adsorption.

The peptide bond yields observed here in the presence of cysteine and achiral silicate minerals were markedly stereoselective with a bias of 50% toward l-cysteine, which is much higher than that reported previously for comparable reaction systems.^17,25,26^ In surface-catalyzed synthesis reactions, stereoselective yields could result from differences in stereoselective surface adsorption, which is possible in the context of naturally chiral surfaces.^27,43,44^ Despite the strong stereoselective bias observed here, we were able to detect covalent silicothioether bonds for both cysteine enantiomers to achiral silicates after extensive washing, suggesting that both enantiomers were strongly adsorbed. Instead, the stereoselective yields observed here are more likely caused by bivalent attachment, which forces enantiomers to adopt different conformations on the silicate surface. Two-point attachment of cysteine via its carboxylate and sulfur groups restricts rotation of the amino acid at the mineral surface, leading to stereospecific differences in the orientation of the amino group, which is the site of peptide bond formation. Reorientation of the amino group could alter solvent accessibility, steric clash, and positioning of the amino group for nucleophilic attack.

A similar stereospecific reorientation of the amino group was observed for aspartate bound to achiral silicates, indicating that it is also able to bind bivalently to silicates. Side chain surface interactions have been proposed previously to affect stereospecific adsorption of aspartate onto chiral surfaces.^43,44^ However, any stereoselective effects of aspartate on the rate of peptide bond formation were not large enough to be detected with achiral silicates under our model solvent conditions. Bivalent attachment of aspartate relies on the carboxylate side chain, which is much less nucleophilic and has a correspondingly weaker silicate association.^33^ In contrast, the cooperativity afforded for cysteine by the extremely strong sulfur attachment coupled to the relatively weaker carboxylate binding means that bivalent attachment and the resulting stereospecific effects can be expected to operate at concentrations significantly lower than those tested here. Additional investigations are required to understand whether such a strong stereoselective bias toward l-cysteine may be amplified and extended to other amino acids incorporated into the growing peptide chain through mechanisms previously described,^45−47^ possibly mediated by stronger peptide surface adsorption or metal ion coordination.

The covalent attachment of cysteine to silicates via the thiol group occurs across a large pH range, and so bivalent attachment and the resulting stereospecific differences in conformation at the surface should be present at any pH greater than the p*K*_a_ of the cysteine carboxylate (>pH 2.0). However, detectable levels of peptide synthesis are restricted to the alkaline pH (>9.50) that is required for amino group deprotonation.^13^ Thus, thiol-mediated stereoselective peptide synthesis is compatible with the alkaline and moderately hot environments required for surface-catalyzed peptide bond formation found in marine “white smoker” hydrothermal vents found on Earth and proposed to exist in the Enceladian ocean.^20^

The addition of cysteine to the reactions also appeared to increase the lengths of the peptides that were produced, but this may be related to increased peptide abundance, which increases the peptide detection probability. In any case, the increase in peptide lengths was modest, suggesting that the presence of cysteine did not significantly overcome the limitation in surface-catalyzed peptide bond formation that peptide adsorption decreases with length.^33^ However, the incorporation of amino acids with thiol-bearing side chains opens the possibility for mechanisms of peptide ligation in solution mediated by metal ion binding.^32,48^

The efficient formation of peptide bonds with cysteine to form thiol-bearing peptides would have been very beneficial in biogenesis, and the use of sulfur-containing methionine to initiate all ribosomal protein synthesis today suggests that sulfur is likely an important ingredient for the earliest stages of life. Cysteines bind metabolically active transition metals such as iron, copper, nickel and cobalt, and also directly mediate oxidation–reduction (redox) reactions in proteins such as thioredoxins.^49^ These thiol-mediated interactions require minimal protein structure, often employing simple sequence motifs, such as CxxC, which are found in some of the most ancient proteins.^50^ Short thiol-bearing peptides have been shown capable of spontaneously ligating metals and exhibiting redox activity,^29,30^ and even free cysteine monomers have been shown to bind and stabilize redox-active FeS clusters.^51^

Our findings indicate that thiol-bearing amino acids strongly increase overall yields in surface-catalyzed peptide bond formation. The yield increases occur with a high stereoselective bias, which may be sufficient to drive symmetry breaking in peptides.^52^ These results add to the growing body of evidence supporting a role for reactive sulfur in abiogenesis and provide further motivation for investigating the availability of cysteine or related thiol-containing amino acids on early Earth, and also whether the presence of such molecules could be a practically useful biosignature for life elsewhere.

## Materials and Methods

### Peptide Synthesis Reactions

Scourie Mòr pyroxenite used as silicate for peptide synthesis reactions was determined from X-ray diffraction (Malvern Panalytical Empyrean) to contain two pryoxene types, Mg-rich enstatite and Ca–Mg-rich diopside, and a serpentine mineral. The pyroxenite was crushed and mesh filtered to less than 106 μm. 50g of the resulting powder was cleaned by washing and drying at 120 °C for 2 h with 2 × 50 mL 60% ethanol (diluted from 100% Stock (ethanol absolute VWR chemicals) and 2 × 50 mL Milli-Q H_2_O across four heat-drying cycles. After cleaning, the pyroxenite was heated for a further 24 h at 200 °C. 0.06 g/mL (of reactant) of cleaned sterilized Scourie *M*_r_ pyroxenite was added into 100 mL borosilicate crimp neck vials (VWR, 548-0609) (50 mL experiments) or 15 mL polypropylene screw cap tubes (SARSTEDT, 62.554.502) (5 mL experiments) to simulate a mineral/ocean interface near a hydrothermal system. For experiments using different oxide minerals, spectroscopy pure SiO_2_, AlO_2_, MgO_2_, and FeO_2_ was added to each reaction vessel as supplied (Sigma-Aldrich).

The ratios of amino acids used were based on the mean rank of relative concentrations of amino acids observed in nonbiological contexts reported by Higgs and Pudritz,^4^ and reaction mixtures were heated in reducing conditions at 90 °C for the times indicated. 20 mM total l- or l + d (1:1) MAAM solutions were prepared from crystalline powders as supplied (>98%, Thermo Fisher Scientific) in the following proportions: glycine (0.41), alanine (0.24), aspartate (0.12), glutamate (0.10), valine (0.04), serine (0.02), isoleucine (0.02), leucine (0.01), proline (0.03), and threonine (0.01). For experiments using isotopically labeled amino acids, crystalline l-cysteine (99% 3-^13^C, Cambridge Isotope Laboratories), glycine ^15^N (98% ^15^N, Sigma-Aldrich), l-methionine (^13^C_methyl_) (Sigma-Aldrich), and l-alanine ^15^N (98% ^15^N, Cambridge Isotope Laboratories) were used. The amino acid mixtures were dissolved in 0.1 M bicarbonate/carbonate buffer (pH 9.55 ± 0.1) that had been prepared from anhydrous sodium carbonate (Fisher Scientific) and sodium bicarbonate (ACS grade, Sigma). Once all amino acids were fully dissolved, 500 mM NH_4_OH was added from a 28–30% NH_4_OH stock solution (ACS grade, 28–30% in water, Thermo Fisher Scientific) and the pH adjusted with 33% HCl or 5 M NaOH to 9.55 ± 0.1.

20 mM stocks of each “excess” amino acid to be tested (>98%, Thermo Fisher Scientific) were prepared separately in 0.1 M bicarbonate/carbonate buffer (pH 9.55 ± 0.1). Solutions contained 20 mM Na_2_S (ACS grade, MP Biomedical) or 20 mM TCEP (Fluorochem) prior to mixing to prevent amino acid oxidation before or after peptide synthesis. The measured redox potential was typically 90–100 mV following synthesis. All solutions were sparged for 20 min in N_2_, followed by a further 30 min equilibration in a 100% N_2_ atmosphere (<10 O_2_ ppm) in a positive pressure anaerobic hood (COY Laboratory Products). Prereaction monomer concentrations were confirmed by measuring the NMR ^1^H–C_α_ peak intensities. Relative concentration differences between l- and d-cysteine stocks were 0.999–1.001.

Following N_2_ equilibration, the MAAM solutions and the excess amino acid solution were added to the borosilicate or polypropylene reaction vessel with 0.06g/mL pyroxene or other oxide mineral. The solutions were diluted with 0.1 M carbonate buffer (pH 9.55 ± 0.1) to a final concentration of 5 mM MAAM, 5 mM TCEP or Na_2_S, 250 mM NH_4_OH, and 5 mM of the excess amino acid. The reaction vessels were crimp sealed or screw capped and heated at 90 °C (Heratherm OGS100).

At reaction completion, the reaction vessels were removed from the oven and uncapped within a fume hood while being cooled to room temperature. Solutions were vacuum pump-filtered through a 0.22 μm poly(ether sulfone) (PES) membrane (MilliPore). The resulting amino acid/peptide mixtures were stored at −80 °C until analyzed by UV-spectrophotometry or lyophilized in 20 or 1 mL aliquots.

### C18 Peptide Purification

Postreaction samples for TIMS-TOF MS were purified by C18 chromatography prior to loading. 4 mL aliquots of the postreaction sample were lyophilized and resuspended into 400 μL of 0.1% trifluoracetic acid (TFA) (Sigma-Aldrich). Separately, C18 SPE tips were prepared by packing 2 μL of C18 resin (Empore SPE C18 48 mm 12 μm particle size) into 10 μL micropipette tips. The resin was activated by washing twice with 60 μL of 100% acetonitrile [high-performance liquid chromatography (HPLC) grade, 99.9%, Sigma-Aldrich], and 60 μL of 0.1% TFA (Sigma-Aldrich) with a 2 min spin at 4000*g* after each addition. 400 μL of the sample in 80 μL aliquots was then pipetted into each SPE tip with a 4 min spin at 5000*g* after each addition. The flowthrough was retained, and the column was washed with 60 μL of 0.1% TFA. Peptides were eluted in 160 μL of 60% acetonitrile in 80 μL aliquots. Elution fractions were lyophilized and stored at 4 °C prior to analysis.

### UV–Vis Spectrophotometry

Postreaction samples in 0.1 M carbonate at pH 9.5 were diluted 1 in 20 with 0.1 M carbonate buffer at pH 9.5 (prepared as described above) to 200 μL. 200 μL of the diluted aliquots was added to an HellmaAnalytics High Precision Quartz SUPRASIL UV–vis cell (light path 10 × 2 mm) and was scanned at wavelengths of 200–800 nm in a Cary 50 Bio UV–visible spectrophotometer with a scan rate of 4800.000 nm/min and 1.0 nm data intervals in dual beam mode with a baseline correction against a 0.1 M carbonate pH 9.5 solution. Three technical repeats were recorded for each sample, and the mean was reported as a single biological repeat for comparison between synthesis runs. The UV–vis cell was cleaned between samples with 1 mL of 2% Hellmanex III (Sigma-Aldrich) and thoroughly rinsed with Milli-Q water between each experiment and dried with N_2_ gas. Biological repeats were normalized relative to that of l-alanine. Results were exported with the absorbance at 230.04 nm plotted for each experiment using GraphPad Prism Software. For measurement of rates of apparent peptide bond formation, concentrations were estimated using 10 mM dialanine (A_230_ = 0.45) as an internal calibration standard.

The statistical significance of differences in UV absorption was determined through one-way ANOVA with Tukey post hoc analysis to determine the effect of each tested excess amino acid on peptide yield compared to an l-alanine negative control.

### Statistical Analysis of UV–Vis Results

The statistical significance of results acquired by UV–vis was determined by one-way ANOVA with Tukey Post Hoc analysis of the mean absorbance values (2–3 technical repeats per sample) of postreactive solutions following heating, as reported for Figure 1a. An unpaired two-tailed *t* test was used instead for data reported in Figure 1b.

### TIMS-TOF MS

TIMS-TOF MS was conducted on purified peptide solutions following C18 SPE purification (described above). 1 μL of purified peptides in 0.1% FA was injected onto a Bruker nanoElute HPLC equipped with a ThermoTrap Cartridge Guard Column (Thermo Scientific 160454) and a Bruker PepSep Fifteen elution column operating at 50 °C (Bruker, 1893473). Peptides were eluted on to a Bruker timsTOF Pro 2 mass spectrometer by gradient elution at a flow rate of 0.55μl/min (Buffer A—0.1% FA, Buffer B—100% ACN 0.1% FA) on a 30 min 0–40% ACN gradient). Detections were analyzed within the Bruker Direct Analysis software. Peak lists were analyzed using a python script (written in house, and available on request), which compared exported mass detections to a list of every amino acid combination of peptides up to 20 amino acids long, and candidate peptides with >5 ppm deviation in mass were discarded. For detections where multiple sequence combinations were possible, the identification with the lowest ppm error with the detected mass was retained. Detections identified also in the negative control were removed. Detections with elution times less than 18 min (≈20% ACN) or greater than 28.5 min (≈38% ACN) and those with signal-to-noise ratios <2, were also removed.

### Solution State NMR

Lyophilized crude or C18 SPE purified postreaction solutions (1 mL original volume) were resuspended into 200 μL of water with 5% D_2_O and 0.4 mM DSS (Cambridge Isotope Laboratories) at pH 5.5 or in 99.9% D_2_O (Sigma-Aldrich) with 5 mM benzoic acid (ACS grade 99.5%, Thermo Fisher Scientific) and 0.4 mM DSS (Cambridge Isotope Laboratories) at pH 5.5. The resulting 5× concentration solutions were loaded into 3 mm Bruker SampleJet NMR tubes. NMR data were recorded in magnets operating at 17.6 or 22.3 T (Oxford Instruments magnets) and equipped with high sensitivity 5 mm TCI cryoprobes and Avance III HD consoles (Bruker). Two-dimensional (2D) ^1^H,^13^C heteronuclear single quantum correlation (HSQC) spectra were acquired with 64 scans, and the total points and sweep widths were 1274 and 320 and 11.934 and 65.00 ppm for ^1^H and ^13^C, respectively. 2D TOCSY data were acquired with 128 scans, sweep widths of 10.000 ppm, and total points of 2048 and 320 in the direct and indirect ^1^H dimensions, respectively. qNMR to measure depletion of monomeric amino acids was performed by integrating and comparing the ^1^Hα signals from monomeric amino acids before and after the reaction. The integrated triplet signal of benzoic acid centered at 7.403 ppm at 20 °C was used as an internal standard to enable quantitative comparison between experimental runs. The results were analyzed with TopSpin 4.3.0.

### Preparation of SiO_2_/Amino Acid Complexes

SiO_2_/amino acid complexes were prepared using the approach of Lopes et al.^42^ Solid amino acid/SiO_2_ samples were prepared in a 100% N_2_ gas atmosphere (<10 ppm of O_2_) to eliminate the requirement for reducing agents during the mixing stage. 500 mM solutions of l- or d-cysteine were prepared by dissolution of crystalline cysteine (>99%; Thermo Fisher Scientific) in Milli-Q water, and the pH was adjusted to 1.5 or 10.5 using 33% HCl or 5 M NaOH. For each reaction, 3.0 g of SiO_2_ (Specpure, Alfa Aesar) was weighed out and placed into a 50 mL polypropylene tube (Sarstedt). Amino acid solutions were added to the SiO_2_ powder, and the tubes were screw-capped. The samples were stirred with a magnetic stir bar for 48 h at 23 °C.

Following mixing, the solutions were vacuum pump-filtered through a 0.22 μm PES membrane (MilliPore) to remove free amino acids. The SiO_2_ solid left on the disk was gently washed and filtered three times with 30 mL of 0.1 M carbonate at pH 10.5 or pH 1.5. Following washing, the SiO_2_ solid was scraped from the membrane and put into a 50 mL polypropylene tube and lyophilized prior to storage at −80 °C before analysis.

### ^13^C Solid State NMR

Solid SiO_2_/cysteine complexes and control powders of free cysteine (99%, Thermo Fisher Scientific) and cystine (99%, Thermo Fisher Scientific) were loaded into a 100 μL rotor. Solid state NMR data were recorded at 9.4 T magnet equipped with an Avance III console (Bruker). ^13^C cross-polarization experiments were recorded with 4096 points and a sweep width of 405 ppm. Data on postreaction samples were recorded with 80,000–100,000 scans, whereas data for the l-cysteine and l-cystine controls were recorded with 64 scans. NMR data were analyzed with TopSpin 4.3.0.

### PR-IRRAS Sample Production and Data Collection

Ti/Au/SiO_2_ microfilm slides mounted on a standard glass slide were prepared at the Thin Film Facility in the Department of Physics, University of Oxford. Glass microscope slides were cleaned prior to coating with Decon 90 and rinsed with deionized water, acetone, and IPA. Slides were loaded into a Leybold L560 and cleaned by Argon glow discharge for 10 min (6 × 10^–2^ mbar). Ti was evaporated using an e-beam (4 × 10^–5^ mbar) and deposited at a rate of 0.3 nm/s to a final thickness of 2 nm. Au was evaporated by thermal evaporation at (2 × 10^–5^ mbar) and deposited at a rate of 0.5 nm/s to 10 nm thickness. Finally, SiO_2_ was evaporated using an ebeam (2.2 × 10^–5^ mbar) and deposited at a rate of 0.25 nm/s to a thickness of 10 nm. Following preparation, the slides were moved to an anaerobic hood and placed at the base of a polypropylene container. Separately, 25 mM solutions of d- and l-aspartate and solutions of 50 mM d- and l-cysteine and d-and l-alanine were made up in Milli-Q water under a 100% N_2_ atmosphere. Solution pH was adjusted to 10.0 using 5 M NaOH and each added to a container with a Ti/Au/SiO_2_ slide. The setup was incubated for 36 h at room temperature. Following incubation, the slides were removed and washed using 20 mL of pH 10.00 degassed Milli-Q water before being dried at room temperature prior to analysis. PR-IRRAS spectra for the Ti/Au/SiO_2_ amino acid complexes were recorded on a Bruker Vertex 80 FT-IR spectrometer equipped with a liquid N_2_ cooled MCT detector and a Pike VeeMax accessory. The spectrometer was referenced to a blank Ti/Au/SiO_2_ slide, and the samples were assessed with s-polarized and p-polarized beams at incidence angles of 30, 60, and 80°. Absolute reflectance of p-polarized beams was plotted using GraphPad Prism software.

## Supporting Information Available

Silicate-dependence of peptide bond yields, ^1^H–^13^C HSQC of reactions with excess ^13^C-labeled methionine, amino acid enrichment in C18 purified peptides, length distribution of synthesized peptides, correlation plot of A_230_ and metal–sulfur bond dissociation enthalpies, comparison of solid state and solution state NMR ^13^C spectra, ^1^H NMR spectra of prereaction mixtures, p-polarized and s-polarized PR-IRRAS spectra of l-cysteine, full PR-IRRAS spectra, statistical analysis of data in Figure 1a, statistical analysis of data in Figure 1b, statistical analysis of data in Figure S4, compositions of peptides detected by TIMS-TOF MS (PDF)
