# Supplementary material for: Bivalent Surface Attachment via Cysteine Thiol Results in Efficient and Stereoselective Abiotic Peptide Synthesis
Source: JACS Au. 2025 Mar 31;5(4):1922–31. doi: 10.1021/jacsau.5c00153 (PMC12041950; doi:10.1021/jacsau.5c00153)
Supplement: Supplementary file 1 — au5c00153_si_001.pdf [file au5c00153_si_001.pdf]

Supporting Information for:

Bivalent surface attachment via cysteine thiol results in efficient and stereoselective abiotic peptide synthesis

Daniel P. Molland[1,*], Isabella B. Rhyu[1], Jon Wade[2,*], Jason R. Schnell[1,*]
[1.] Department of Biochemistry, University of Oxford, South Parks Road, OX1 3QU
[2.] Department of Earth Sciences, University of Oxford, South Parks Road, OX1 3AN, UK

***Corresponding Authors:**
Daniel.Molland@sjc.ox.ac.uk, Jon.Wade@earth.ox.ac.uk, Jason.Schnell@bioch.ox.ac.uk

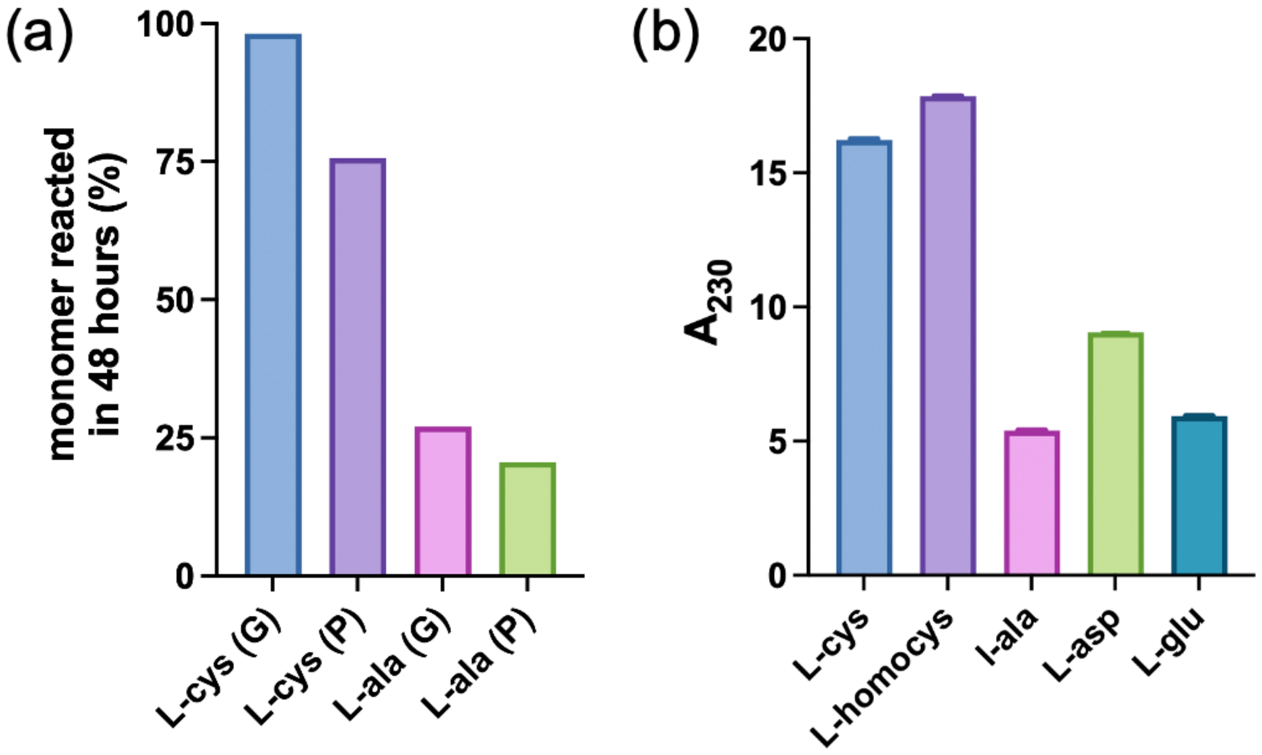

Figure S1: The presence of a free thiol side chain significantly increases peptide yield in the presence of silicate.

(a) The amount of monomeric amino acid reacted after 48 hours in the peptide synthesis reaction containing an excess of L-alanine or L-cysteine in borosilicate glass (G) or polypropylene (P) reaction vessels. The amino acids were measured by Quantitative NMR using a benzoic acid internal standard. Error bars display standard deviation of the benzoic acid signal intensity at 7.4034 ppm.

(b) Peptide abundance as determined by A_{230} for reactions carried out in borosilicate reaction vessels and pyroxenite with a 5mM excess of the indicated amino acids. Error bars display the standard deviation of instrument noise at 400-800nm.

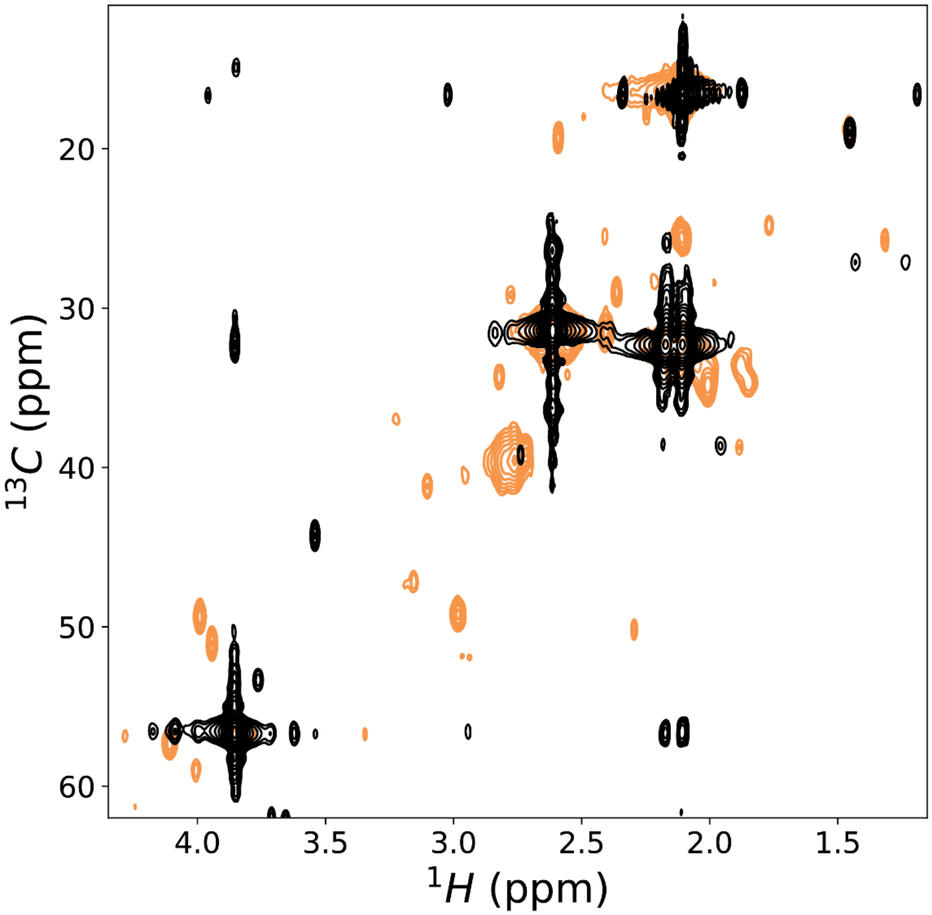

Figure S2: Peptide synthesis in the presence of excess methionine produces a low diversity of products.

^{1}H-^{13}C HSQC of reactions with excess ^{13}C-labelled methionine. Reactions were set up under identical conditions to those in **Figure 2a** and carried out in borosilicate reaction vessels for 48 hours at 90˚C.

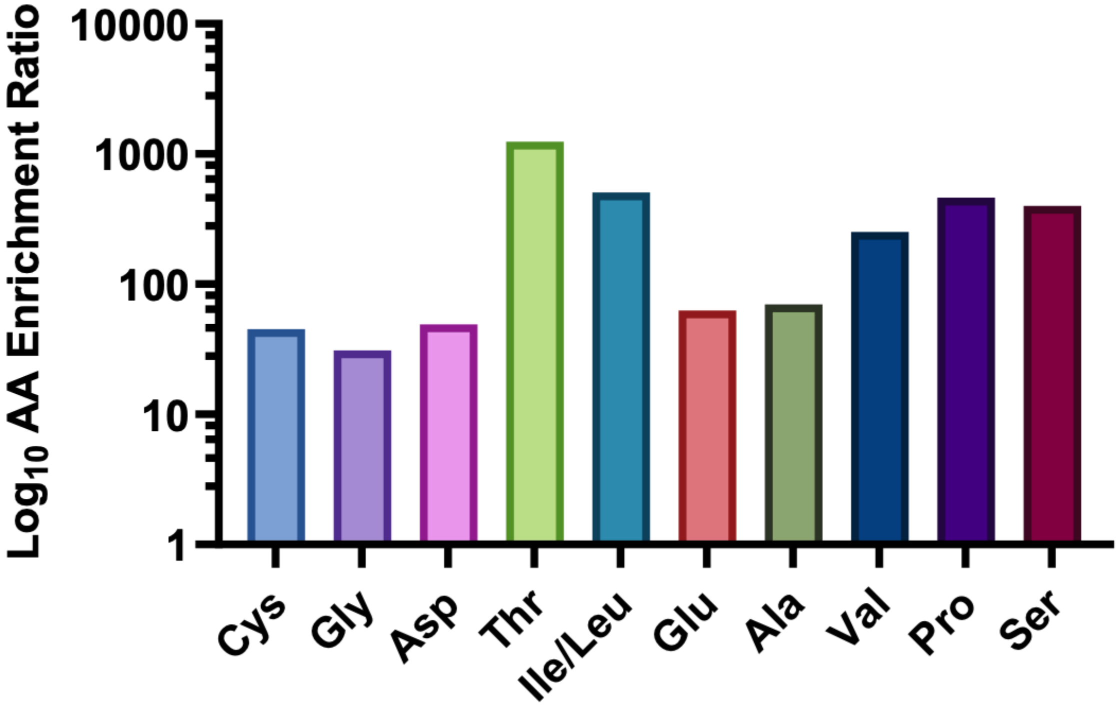

Figure S3: Hydrophobic amino acids are enriched in peptides C18 SPE purified and detected by TIMS-TOF MS.

Peptide reaction mixtures were C18 SPE purified and analysed by TIMS-TOF MS. Amino acid enrichment was determined by dividing the observed amino acid abundance in the detected peptides by the relative abundance added to the reaction mixtures.

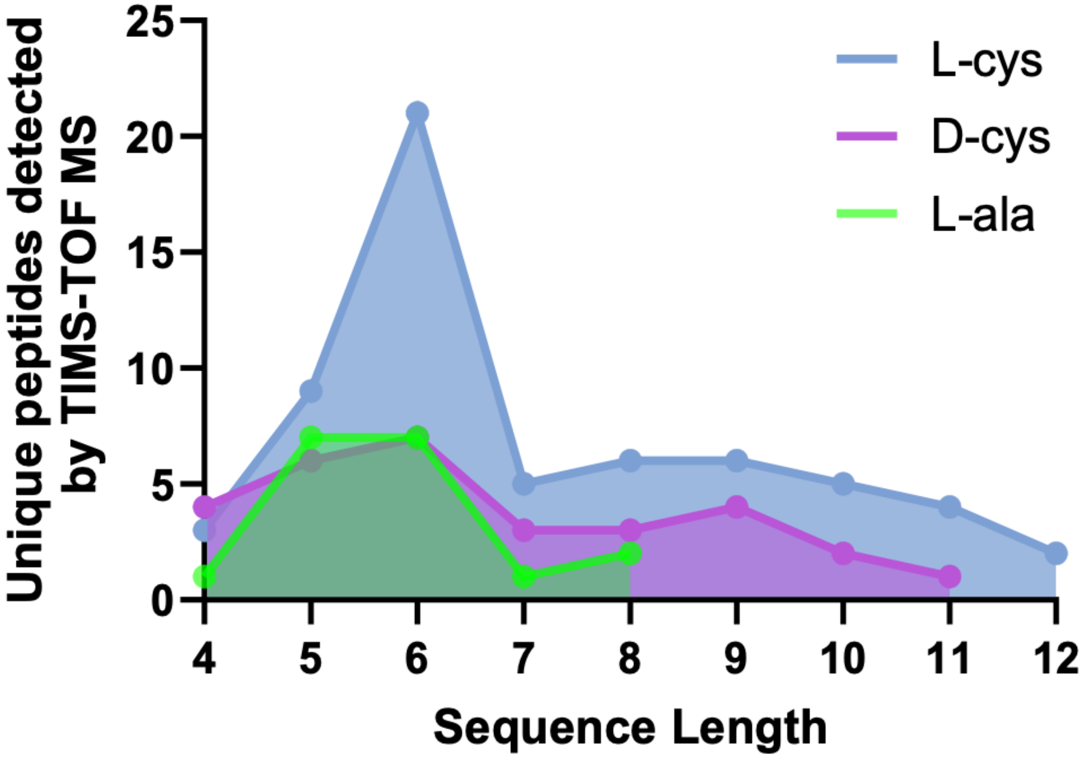

Figure S4: Synthesis with L-cysteine increases peptide yield and the length of detected peptides. The distribution of peptide lengths for reactions in the presence of excess L-cysteine or L-alanine for peptides detected by TIMS-TOF MS. Peptides were synthesised with excess L-cysteine, D-cysteine and L-alanine, and then C18 SPE purified and analysed by TIMS-TOF MS.

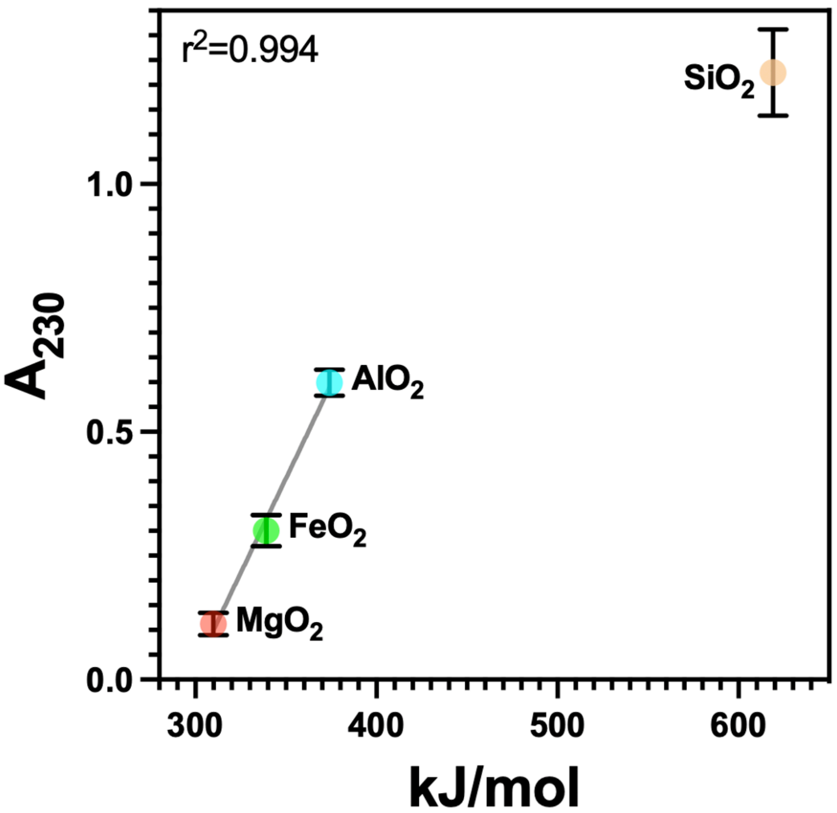

Figure S5: Abiotic peptide bond formation in the presence of L-cysteine correlates with metal-sulphur bond dissociation enthalpies.

Peptides were synthesised as described in polypropylene reaction vessels with different silicate minerals present for 2 days. Peptide formation was assessed by A_{230} and metal-sulphur bond enthalpies are taken from Cottrell et al. (1958)[40]. Error bars display the standard deviation of instrument noise at 400-800nm. The linear regression fit and correlation coefficient shown were calculated for the metal-containing silicates only.

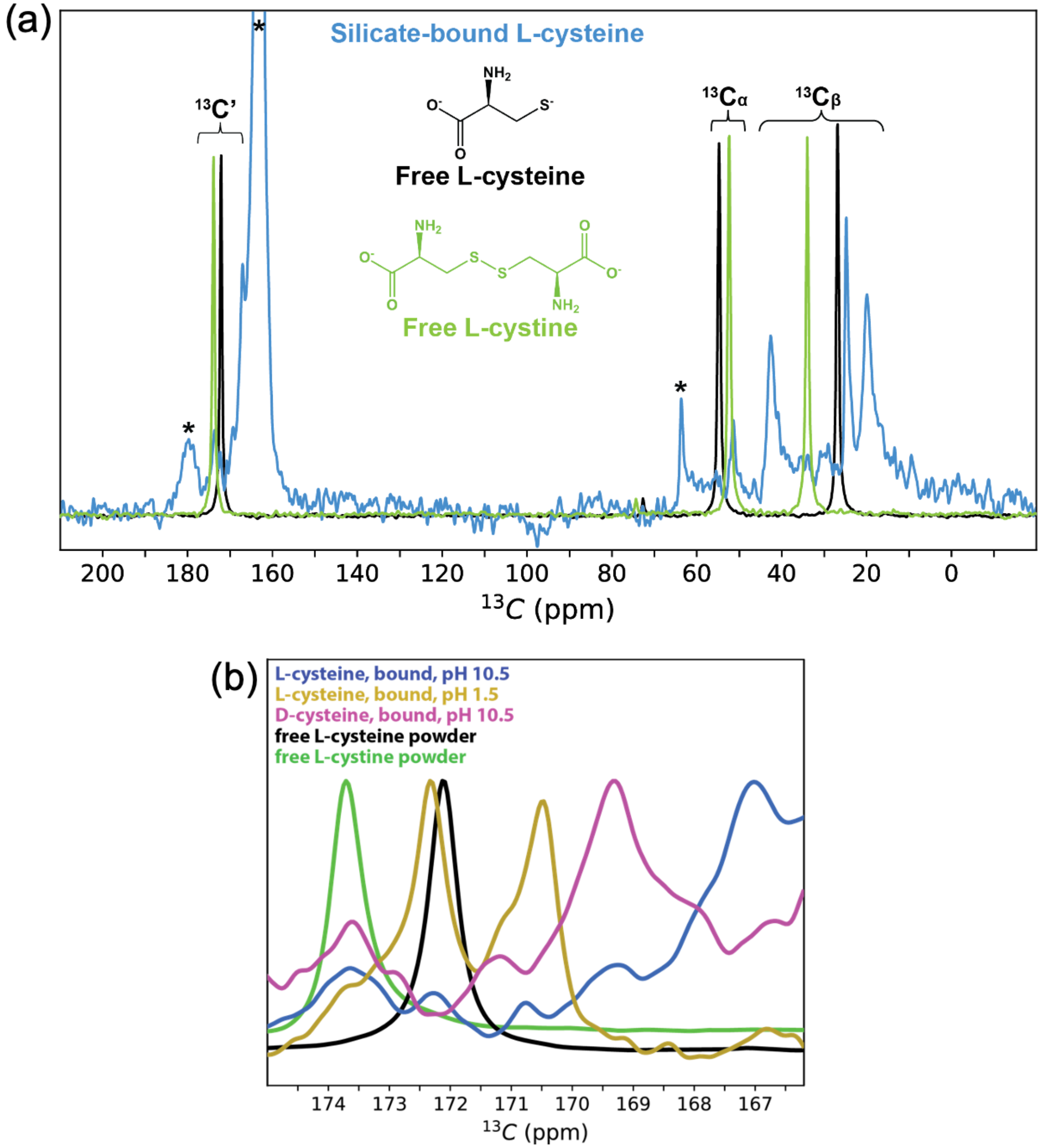

Figure S6: Comparison of the ^{13}C solid-state NMR spectra of silicate-bound L-cysteine and free L-cysteine and free L-cystine.

(a) Solid state NMR ^{13}C cross polarisation spectra of L-cysteine attached to silicate (blue) compared with free L-cysteine powder (black) and free L-cystine powder (green). The regions corresponding to the carboxylate ^{13}C', ^{13}Cα, and ^{13}Cβ signals are indicated. Wash buffer signals at 163.2ppm (bicarbonate), 169.3ppm (carbonate) 179.7ppm (carbonate salt), and 63.7ppm (bicarbonate sideband) are indicated (*) **(b)** The carboxylate region of the solid state NMR ^{13}C cross polarisation spectra.

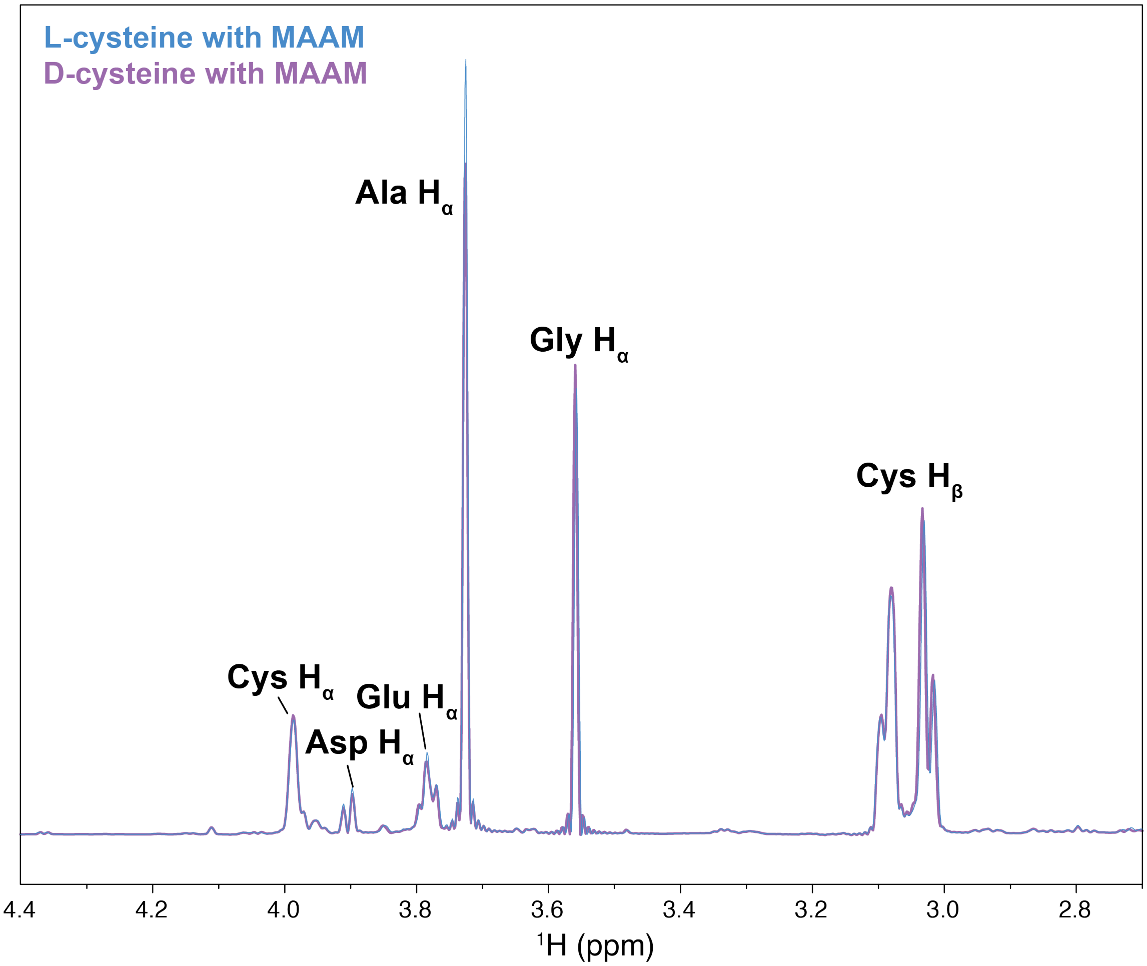

Figure S7: ^{1}H NMR of pre-reaction mixtures with excess L-cysteine or D-cysteine.
Confirmation of the chemical shift redundancy of the signals from L-cysteine and D-cysteine in solution. Labels indicate ^{1}H signals from the most abundant amino acids.

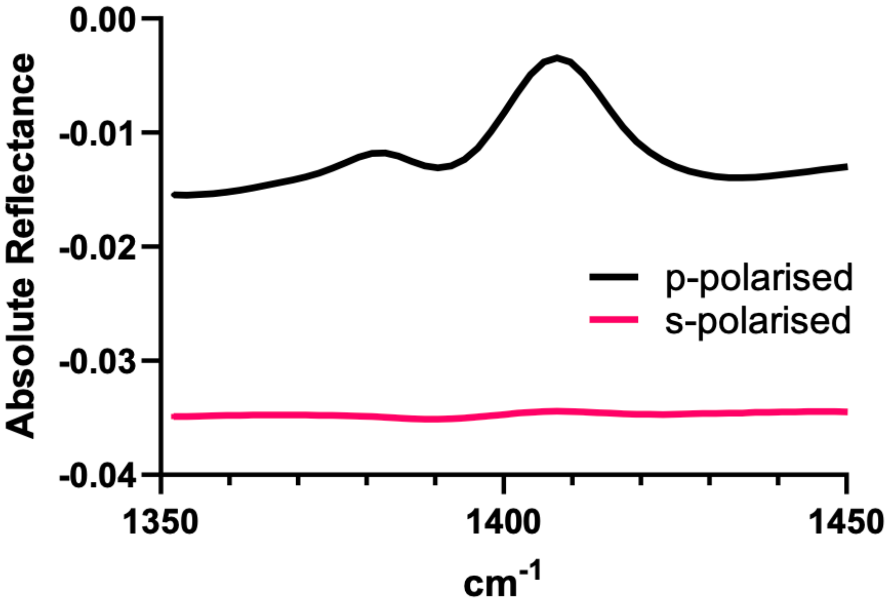

Figure S8: A covalent bond between the cysteine thiolate and silicate is detected by PR-IRRAS. PR-IRRAS spectra of L-cysteine attached to silicate produces a reflectance band for p-polarised light at 1407.2 cm^{-1}, consistent with a silicothioether bond[44]. No reflectance is observed for s-polarised light indicating that the reflectance for p-polarised light arises from molecules at the silicate surface.

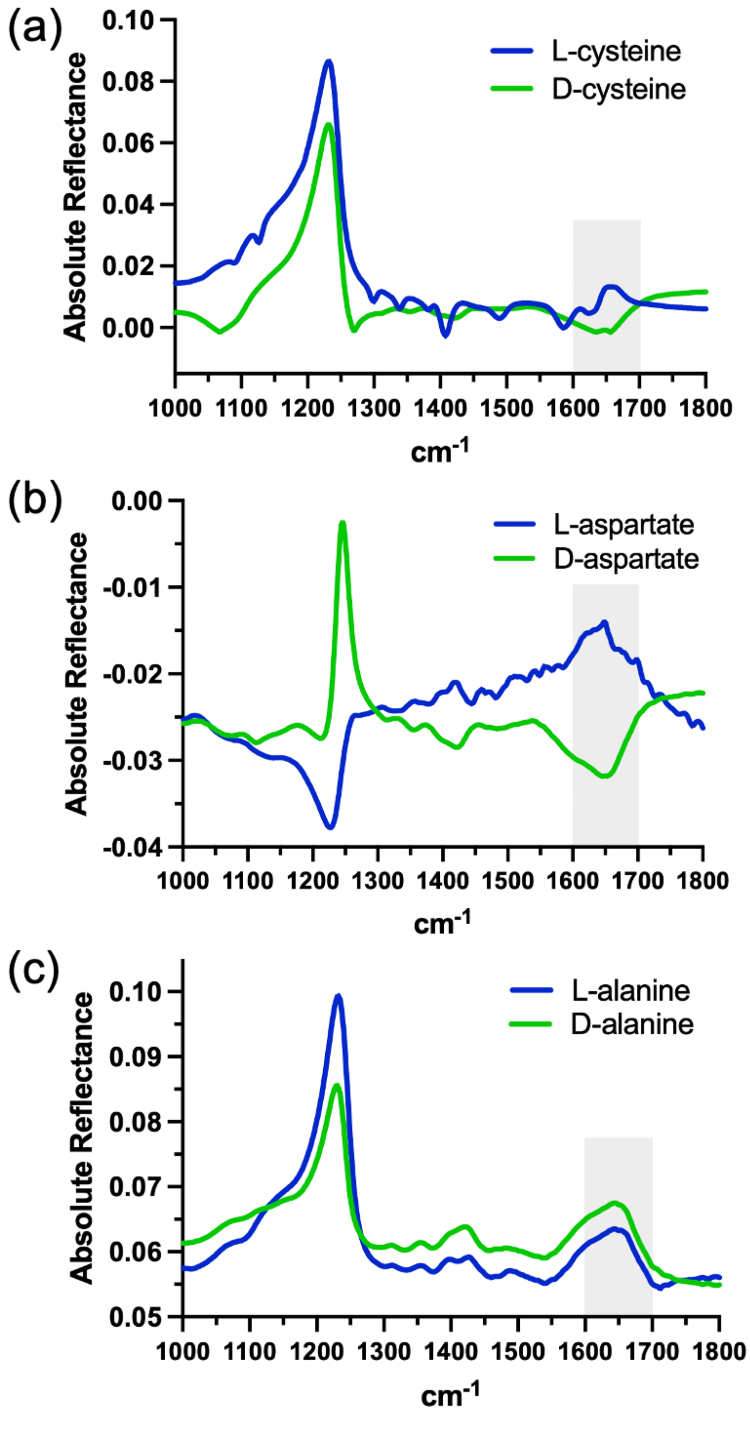

Figure S9: Full PR-IRRAS spectra of the cysteine, aspartate and alanine enantiomers absorbed to silicate.

PR-IRRAS of **(a)** cysteine, **(b)** aspartate, and **(c)** alanine enantiomers absorbed to silicate. Stereo-specific band inversions in the region corresponding to the asymmetric bend of the amino group (~1600-1700 cm^{-1}) were observed for cysteine and aspartate enantiomers but not alanine enantiomers.

Table S1: Statistical analysis of data in Figure 1a

Statistical Test: One Way ANOVA with Tukey Post Hoc Analysis			
F		29.05	
P value		<0.0001	
R squared		0.8994	
Tukey's multiple comparisons test	Mean Diff.	95.00% CI of diff.	Adjusted P Value
L-cys vs. D-cys	8.260	3.405 to 13.12	0.0006
L-cys vs. L-ala	12.67	8.041 to 17.30	<0.0001
L-cys vs. D-ala	15.02	9.350 to 20.69	<0.0001
L-cys vs. L-met	15.44	8.893 to 21.99	<0.0001
D-cys vs. L-ala	4.410	-0.4452 to 9.265	0.0851
D-cys vs. D-ala	6.760	0.9044 to 12.62	0.0196
D-cys vs. L-met	7.180	0.4716 to 13.89	0.0328
L-ala vs. D-ala	2.350	-3.320 to 8.020	0.7172
L-ala vs. L-met	2.770	-3.777 to 9.317	0.7021
D-ala vs. L-met	0.4200	-6.899 to 7.739	0.9998

Table S2: Statistical analysis of data in Figure 1b

Statistical Test: Unpaired T Test	
P value	0.0015
P value summary	**
One- or two-tailed P value?	two-tailed
t, df	t=7.696, df=4

Table S3: Statistical analysis of data in Figure S4

Statistical Test: Mann Whitney Test	
L-cysteine vs L-alanine	
P value	0.0188
P value summary	*
T Test Type	Two-tailed
Sum of Ranks	111.50, 59.50
Mann Whitney U	14.50
L-cysteine vs D-cysteine	
P value	0.0946
P value summary	ns
T Test Type	Two-tailed
Sum of Ranks	104.50, 66.50
Mann Whitney U	21.50
L-alanine vs D-cysteine	
P value	0.1563
P value summary	ns
T Test Type	Two-tailed
Sum of Ranks	69.50, 101.50
Mann Whitney U	24.50

Table S4: Compositions of peptides detected by TIMS-TOF MS. Peptides were synthesised in the presence of excess L-cysteine or L-alanine and heated at 90˚C for 42 days, C18 purified, and analysed by TIMS-TOF MS (see Supplementary Methods 'Peptide Synthesis Reactions' and 'TIMS-TOF MS'). Amino acid compositions (alphabetical) and theoretical masses of peptides are indicated. Cysteine bearing sequences are in red text.

Excess L-cysteine			
**Detected peptide composition**	**Detected mass**	**Predicted mass**	**Detection ppm error**
ADG	262.1023	262.1034	3.5553
TTT	322.1614	322.1609	1.0486
CDS	324.0844	324.0860	4.5309
IIS	332.2191	332.2180	2.6076
ACEG	379.1295	379.1282	2.8087
CCEI	467.1649	467.1629	3.8654
DEEE	521.1702	521.1726	4.0416
AVVVV	486.3274	486.3286	1.8722
AADDT	492.1918	492.1936	3.1741
ACCDT	512.1476	512.1479	0.0558
ADDPT	518.2080	518.2093	1.8409
EPSST	520.2253	520.2249	0.1170
CCCTT	530.1401	530.1408	0.7533
AEIIP	542.3197	542.3185	1.8176
CEEGI	550.2181	550.2178	0.1540
EIIPP	568.3325	568.3341	2.3743
AAADGV	503.2440	503.2460	3.3884
ACCGGP	507.1698	507.1690	0.9598
ADGGPT	517.2250	517.2253	0.0396
AAACPT	533.2375	533.2388	1.9510
CGPSSS	537.1978	537.1973	0.2772
AGPPVV	539.3169	539.3188	3.0230
AAADPP	541.2613	541.2616	0.1872
AEEGGS	549.2152	549.2151	0.4262
CEEGGG	551.1766	551.1766	0.5475
CCGSST	557.1700	557.1694	0.4887
CEGPSS	579.2082	579.2079	0.0711
APPTVV	583.3427	583.3450	3.4133
CCCCGP	585.1266	585.1288	3.1920
PPPSST	585.2880	585.2879	0.3962
CCCPSS	599.1640	599.1622	2.4009
STTTTV	609.3088	609.3090	0.1610
CIPPSV	615.3191	615.3171	2.7594
CCDSTV	627.2083	627.2113	4.1426
EIPPSS	629.3145	629.3141	0.0348
CCCIPP	635.2375	635.2350	3.3243
CCCDIP	653.2115	653.2092	2.9513
CDGGGGP	562.1912	562.1926	1.8791

AACCCCD	688.1540	688.1557	1.9967
CCCCCTT	736.1630	736.1591	4.7475
CCIIIVV	762.4263	762.4252	0.7770
DDIIPPP	766.3981	766.3982	0.4534
AAAACGGG	577.2370	577.2399	4.4542
GGISSSST	695.3234	695.3206	3.4266
CEEGGGPP	745.2834	745.2821	1.1371
CDDGSSST	771.2436	771.2461	2.7157
EIIPPSSS	829.4303	829.4302	0.4318
CIIIIPPT	869.5151	869.5165	1.0804
GGGGGPPPT	696.3274	696.3311	4.8569
AAAAAPVVV	768.4579	768.4614	4.0371
AAGVVVVVV	812.5210	812.5240	3.1914
CCCCCGGPS	832.1876	832.1915	4.1571
AADPVVVVV	868.5109	868.5138	2.8593
CCCDIIIII	1008.4930	1008.4930	0.3972
AAGGGGGGPP	697.3237	697.3264	3.3004
CCCGGGGGGV	769.2440	769.2426	1.3260
AAAAAAADVV	829.4410	829.4414	0.1075
AAADGTTVVV	903.4771	903.4782	0.6410
CCCCCCGGGP	905.1857	905.1901	4.2720
AAAAAAAAAAT	830.4337	830.4366	3.0011
GGGGPPPPPPP	926.4718	926.4731	0.7798
ACCCCCCCGGT	1026.2060	1026.2100	2.8299
AACDDVVVVVV	1088.5640	1088.5660	0.8864
AAACGGGGGPPP	911.4030	911.4040	0.4974
AADDGTTTTTTV	1153.5220	1153.5220	0.2206

Excess L-alanine			
Detected peptide composition	Detected mass	Predicted mass	Detection ppm error
DIII	473.2954	473.2970	2.7714
AGIIT	474.2923	474.2922	0.5142
DGVVV	488.2697	488.2715	3.1303
DSVVV	518.2808	518.2821	1.9660
TTTVV	520.2976	520.2977	0.3136
IISTT	534.3142	534.3134	1.0652
EPPPP	536.2692	536.2715	3.6941
EIITT	576.3223	576.3239	2.3720
GGPPPS	511.2512	511.2511	0.3309
GPPPPV	563.3165	563.3188	3.5258
DDDGGP	575.1943	575.1944	0.4335
ADPPTV	599.3032	599.3035	0.0323
ISSTTT	609.3093	609.3090	0.0658
IITPPP	637.3899	637.3920	2.7604
DDIPVV	657.3454	657.3454	0.5074
AAAPVVV	626.3852	626.3872	2.5679

AAAAAAAV	615.3436	615.3460	3.3372
GGGGPSST	619.2716	619.2682	4.8790

Excess D-cysteine			
**Detected peptide composition**	**Detected mass**	**Predicted mass**	**Detection ppm error**
ET	249.1091	249.1081	3.4402
ADG	262.1022	262.1034	3.7842
CGPP	373.1536	373.1540	0.6898
ADPP	399.1858	399.1874	3.6510
EPPT	443.2113	443.2137	4.7296
DIIP	457.2654	457.2657	0.1695
AAPPP	452.2494	452.2504	1.6921
AVVVV	486.3268	486.3286	3.2293
PPPPT	508.2764	508.2766	-0.2608
ACCDE	540.1402	540.1429	4.4103
DIIVV	558.3488	558.3498	1.1156
CDDEP	578.1792	578.1763	4.5148
GGGGIT	461.2361	461.2354	0.8354
GGGPPS	471.2175	471.2198	4.2758
ADGGPT	517.2247	517.2253	0.5791
AAACCD	553.1744	553.1745	-0.4700
PPSSSS	561.2517	561.2515	-0.1669
STTTTV	609.3079	609.3090	1.1848
CDPSVV	619.2725	619.2756	4.4908
GGGGGPS	488.2126	488.2100	4.7694
GGGGGII	530.2954	530.2933	3.4365
CIISSSS	696.3269	696.3233	4.5976
GGISSSST	695.3238	695.3206	4.0306
CGISSSSS	727.2951	727.2927	2.7380
GGIIISSS	733.4131	733.4091	4.9353
GGGGIIPST	758.4069	758.4043	2.8677
AAAAAPVVV	768.4581	768.4614	3.6987
AAGVVVVVV	812.5204	812.5240	3.8806
AGTVVVVVV	842.5317	842.5346	2.9122
AAGGGGGGPP	697.3231	697.3264	4.0891
CDGGGGPPSS	833.3118	833.3094	2.2909
ACCGGGGGGPP	832.3084	832.3076	0.4193
